# Angiotensinogen Suppression: A New Tool to Treat Cardiovascular and Renal Disease

**DOI:** 10.1161/HYPERTENSIONAHA.122.18731

**Published:** 2022-07-29

**Authors:** Edwyn O. Cruz-López, Dien Ye, Congqing Wu, Hong S. Lu, Estrellita Uijl, Katrina M. Mirabito Colafella, A.H. Jan Danser

**Affiliations:** Division of Pharmacology and Vascular Medicine, Department of Internal Medicine, Erasmus MC, University Medical Center Rotterdam, the Netherlands (E.O.C.L., D.Y., E.U., A.H.J.D.).; Saha Cardiovascular Research Center (C.W., H.S.L.), University of Kentucky.; Department of Surgery (C.W.), University of Kentucky.; Department of Physiology (H.S.L.), University of Kentucky.; Monash University, Melbourne, Australia (K.M.M.C.).

**Keywords:** angiotensin, angiotensinogen, hypotension, oligonucleotides, antisense, renin

## Abstract

Multiple types of renin-angiotensin system (RAS) blockers exist, allowing interference with the system at the level of renin, angiotensin-converting enzyme, or the angiotensin II receptor. Yet, in particular, for the treatment of hypertension, the number of patients with uncontrolled hypertension continues to rise, either due to patient noncompliance or because of the significant renin rises that may, at least partially, overcome the effect of RAS blockade (RAS escape). New approaches to target the RAS are either direct antisense oligonucleotides that inhibit angiotensinogen RNA translation, or small interfering RNA (siRNA) that function via the RNA interference pathway. Since all angiotensins stem from angiotensinogen, lowering angiotensinogen has the potential to circumvent the RAS escape phenomenon. Moreover, antisense oligonucleotides and small interfering RNA require injections only every few weeks to months, which might reduce noncompliance. Of course, angiotensinogen suppression also poses a threat in situations where the RAS is acutely needed, for instance in women becoming pregnant during treatment, or in cases of emergency, when severe hypotension occurs. This review discusses all preclinical data on angiotensinogen suppression, as well as the limited clinical data that are currently available. It concludes that it is an exciting new tool to target the RAS with high specificity and a low side effect profile. Its long-term action might revolutionize pharmacotherapy, as it could overcome compliance problems. Preclinical and clinical programs are now carefully investigating its efficacy and safety profile, allowing an optimal introduction as a novel drug to treat cardiovascular and renal diseases in due time.

Worldwide 1 in 3 adults are hypertensive. Although the treatment of hypertension effectively prevents death and disability, the number of patients with uncontrolled hypertension continues to rise.^1,2^ This may be attributed, at least partly, to patient noncompliance, that is, the tendency for patients to take their medication intermittently or stop taking them completely,^3^ rather than treatment-resistant hypertension. The renin-angiotensin system (RAS) plays a pivotal role in the long-term regulation of blood pressure and is a mainstay for the treatment of cardiovascular and kidney diseases. Ang (angiotensin) II, the main effector peptide of the RAS, stimulates the AT_1_ (Ang II type 1) receptor to elicit all the classical actions of the RAS, including vasoconstriction, water and sodium retention, aldosterone synthesis, proinflammatory effects, and growth and remodeling. Although several drug classes already exist to target the RAS, for example, direct renin inhibitors, ACE (angiotensin-converting enzyme) inhibitors, AT_1_ receptor blockers (ARB) as well as the dual AT_1_ receptor neprilysin inhibitor (Figure 1), there remains an unmet need for a RAS inhibitor that can circumvent the so-called RAS escape phenomenon and issues pertaining to patient noncompliance. This RAS escape concerns the fact that Ang II suppresses renin release via its AT_1_ receptor (the so-called negative feedback loop), implying that any type of RAS blockade will prevent this suppression, so that renin will rise, thereby potentially overcoming the effect of RAS blockade. As an example, a direct renin inhibitor capable of blocking renin by 90% would require a renin rise of 10-fold to bring back Ang I generation to its predirect renin inhibitor level. Given that in humans renin rises of several 100-fold are feasible,^4^ the capacity of the body to overcome RAS blockade is indeed substantial and is likely to diminish the net effect of RAS blockade. The development of novel RAS inhibitors that target liver-derived AGT (angiotensinogen) has the potential to avoid these problems, thereby improving patient outcomes and reducing the global burden of disease.

**Figure 1.**
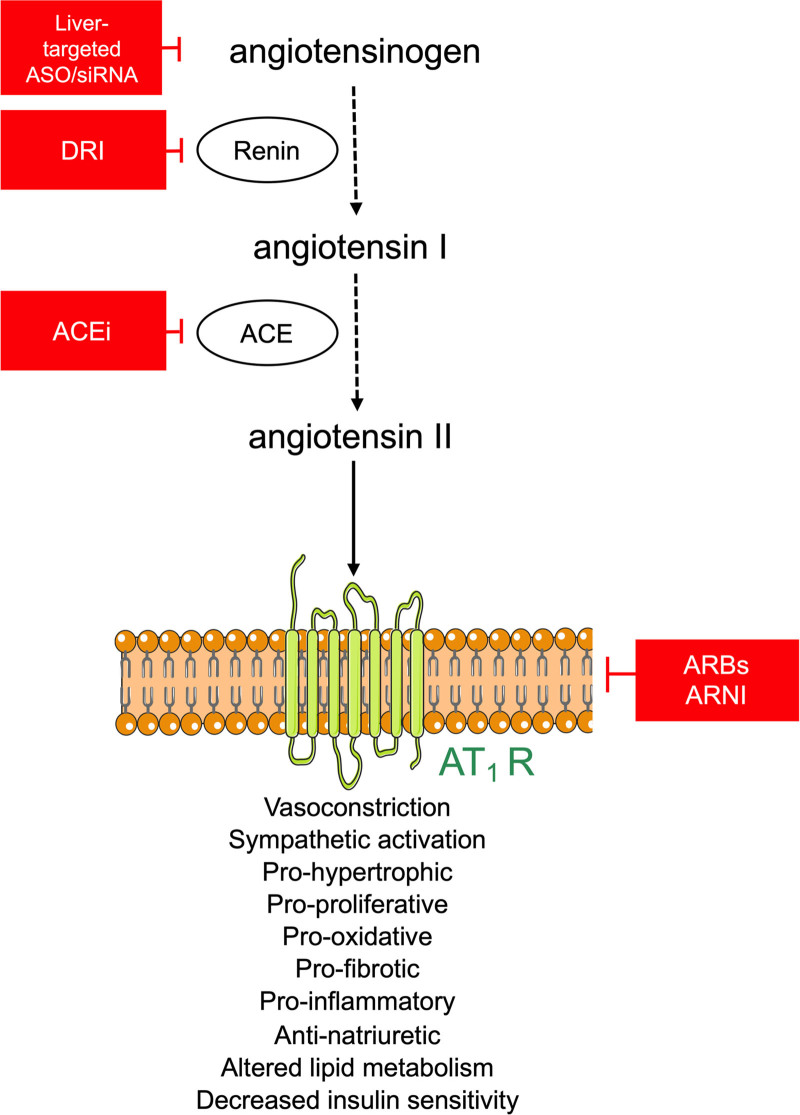
**The renin angiotensin system and its therapeutic targets.** ACE indicates angiotensin-converting enzyme; ACEi, ACE inhibitor; ARBs, angiotensin II type 1 receptor blockers; ARNI, angiotensin receptor neprilysin inhibitor; ASO, antisense oligonucleotides; AT_1_R, angiotensin II type 1 receptor; DRI=direct renin inhibitor and siRNA, small interfering RNA.

AGT, the substrate for renin, is the source of all angiotensin peptides (Figure 1). AGT is primarily synthesized by the liver, stimulated by ethinylestradiol, dexamethasone, and T3^5^; however, other tissues, including the brain,^6,7^ adipose tissue,^5,8^ and kidney,^9–11^ are proposed to synthesize AGT as well. Accordingly, it has been suggested that the regulation and functioning of autonomous local or tissue RAS in the kidney, as well as other organs, may contribute to the activity of the system in physiological and pathophysiological situations.^12,13^ This hypothesis has been used to explain the complexity of the system, whereby the apparent activity of the RAS is not reflected by measured plasma levels of its major components. Yet, studies utilizing liver-specific *AGT* gene deletion in mice,^14^ and more recently, the use of novel hepatocyte-directed ribonucleic (RNA)-based therapies to inhibit the formation liver-derived AGT in rats and nonhuman primates,^15–18^ have been instrumental in demonstrating that circulating, adipose tissue and kidney AGT is derived primarily from synthesis in the liver.

Several approaches have been developed to target liver-derived AGT. Two such approaches utilize trivalent N-acetylgalactosamine (GalNAc)-conjugation to direct antisense oligonucleotides (ASO) that inhibit RNA translation and small interfering RNA (siRNA) that function via the RNA interference pathway towards the liver (Figure 2). GalNAc binds to the asialoglycoprotein receptor that is highly expressed on hepatocytes and results in rapid endocytosis.^19^ In brief, the ASO approach utilizes single-stranded DNA which binds to its target mRNA, with RNase H1 cleaving the RNA in an RNA-DNA duplex in both the cytoplasm and nucleus,^20^ thereby destroying the mRNA and inhibiting the translation of the target protein, that is, AGT. In contrast to the ASO approach, siRNAs are delivered as duplexes which are then taken up by Argonaute, part of the RNA-induced silencing complex. When siRNA enters the cell it stays inactive until loaded into Argonaute where the passenger strand is removed and the remaining antisense strand binds to complementary mRNA targets, leaving Argonaute endoribonuclease to cleave the mRNA,^21^ thus preventing protein translation. In preclinical models, GalNAc-conjugation has been shown to enhance ASO delivery to hepatocytes by ≈10-fold versus free ASOs,^22^ whereas in siRNA-treated animals, hepatic siRNA levels were ≈50-fold higher than that found in the kidney.^17,18^ Although both GalNAc-conjugated siRNA and ASO targeting liver-derived AGT have been shown to lower blood pressure in spontaneously hypertensive rats (SHR),^17,18^ the latter approach typically requires weekly dosing and may have a less than ideal safety profile.^17,18,23^ In contrast, storage of siRNA in hepatic endosomes ensures prolonged target knockdown that lasts for weeks to months after a single subcutaneous injection.^24^ In a phase 2 clinical trial, hepatocyte-directed GalNAc-siRNA molecules have been shown to yield incredible results with stable suppression of PCSK9 (proprotein convertase subtilisin/kexin type 9) for at least 6 months.^25^ In rodents, we have demonstrated that GalNAc-conjugated siRNA suppresses liver AGT for at least 2 weeks.^16–18^ Furthermore, since GalNAc-conjugated siRNA leaves little AGT and the remainder is depleted due to accelerated consumption by the compensatory rise in renin, the reconstitution of Ang II appears finite,^17^ suggesting that this approach confers resistance to the RAS escape phenomenon. Currently, the GalNAc-conjugated ASO (IONIS-AGT-L_Rx_, Ionis Pharmaceuticals) phase I/II clinical trials have been completed,^26^ and GalNAc-conjugated siRNA (Zilebesiran [ALN-AGT], Alnylam Pharmaceuticals) phase I/II clinical trials will be completed soon. The third approach to inhibit the formation of liver-derived AGT is to utilize gene editing technology, such as the clustered regularly interspaced short palindromic repeats/ clustered regularly interspaced short palindromic repeat-associated protein 9 (CRISPR/Cas9) gene editing system recently proposed by Sun et al.^27^ In contrast to the RNA-based therapeutics which inhibit the formation of liver-derived AGT for weeks to months, this approach results in the permanent disruption of the target gene, that is, it is irreversible. Although this approach is still in its infancy, initial studies in SHR suggest that partial deletion (40%) of the hepatic *AGT* gene is sufficient to prevent the development of hypertension in this model.^27^

**Figure 2.**
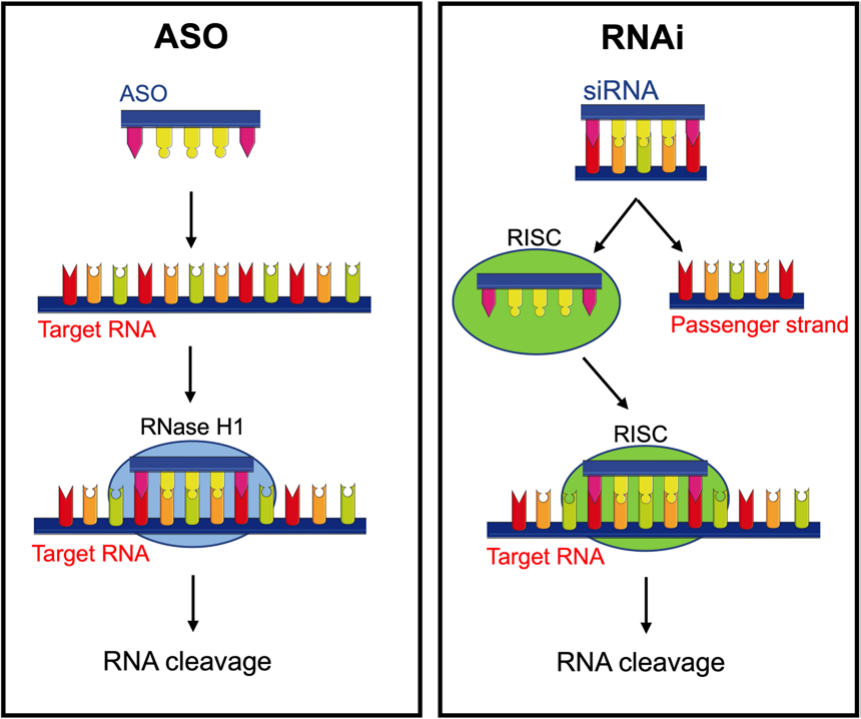
**Schematic of the RNA-based approaches used to target liver-derived AGT (angiotensinogen).** Antisense oligonucleotides (ASOs) bind to their targeted RNA and use endogenous RNase H1, an enzyme that leads to RNA cleavage. The RNA interference (RNAi) utilizes the RNAi-induced silencing complex (RISC) containing a small interfering RNA (siRNA) to specifically degrade the targeted RNA.

Chronically inhibiting the RAS by targeting liver-derived AGT has several advantages over currently available RAS inhibitors; however, it is not without its limitations. Selectively deleting liver-derived AGT may limit the body’s ability to respond to physiological challenges since activation of the RAS plays an important role in correcting both acute and chronic hemodynamic changes including hemorrhage and more slowly developing hypovolemia such as dehydration as well as facilitating the normal cardiovascular and renal adaptations to pregnancy. Here we discuss the potential benefits of selectively deleting liver-derived AGT for cardiovascular and kidney diseases and consider the side effects which may limit its broad therapeutic application.

## AGT Suppression in Hypertension

**Figure 3.**
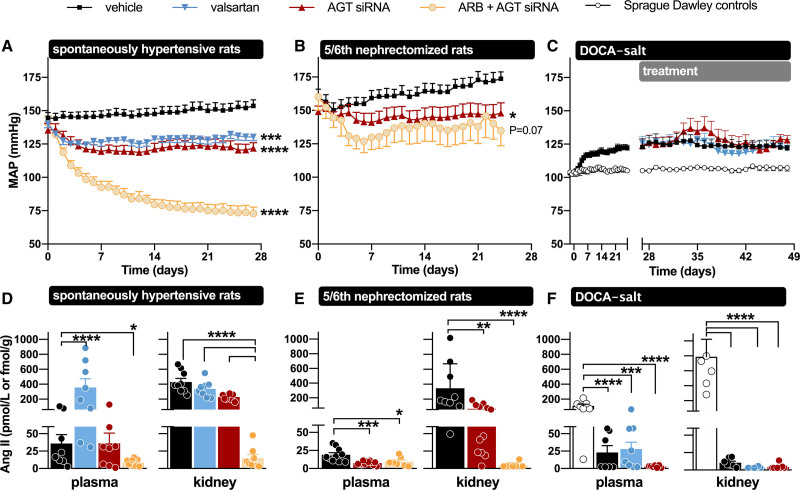
**Overview of the effects of AGT (angiotensinogen) small interfering RNA (siRNA), Ang (angiotensin) II type 1 receptor blockade, or their combination on blood pressure, circulating Ang II, and renal Ang II in various hypertensive rat models.** In the spontaneously hypertensive rats and the deoxycorticosterone acetate (DOCA) salt rats, valsartan was used, while in the 5/6th nephrectomy rats losartan was used. Ang indicates angiotensin; ARB, Ang II type 1 receptor blockers; DOCA, deoxycorticosterone acetate; and MAP, mean arterial blood pressure. *P<0.05, **P<0.01, ***P<0.001, ****P<0.0001. Data have been modified from Uijl et al. and Bovée et al.^16–18^

Both the ASO and siRNA approach targeting AGT are capable of lowering circulating AGT in SHR (Figure 3).^17,28^ Lowering occurred in a dose-dependent manner, and GalNAc-conjugation substantially increased potency. The degree of lowering that could be reached was >99.5%. Non-GalNac–conjugated ASOs also suppressed extrahepatic AGT expression, for example, in kidney and adipose tissue, whereas GalNac-conjugation, as expected, exclusively resulted in hepatic AGT suppression.^28^ Given the fact that circulating AGT lowering was identical with free and GalNAc-conjugated ASO/siRNA, it can be concluded that circulating AGT is liver-derived, in full agreement with data in liver AGT knockout animals.^29,30^ This implies that AGT synthesis at extrahepatic sites, if occurring, allows local angiotensin generation only.

Remarkably, lowering circulating AGT by 97.9% with GalNAc-conjugated AGT siRNA did not significantly lower circulating Ang II (Figure 3).^17^ This was due to the renin rises that accompany this approach, which are apparently sufficient to restore Ang II to its pretreatment levels. Here it is important to stress that plasma AGT levels in rats, like in humans, are in the 1 µmol/L (ie, K_m_) range,^31^ while circulating Ang II levels are in the low pmol/L range.^32^ In other words AGT levels are easily 5 to 6 orders of magnitude above those of Ang II. Even when lowering circulating AGT by 99%, they are still 3 to 4 orders of magnitude above those of Ang II. In fact, this situation exists normally in mice. Mice display AGT levels which are at most a few percent of those in rats in humans.^33^ Nevertheless, mice have Ang II levels that are identical to those in rats and humans,^34–36^ which is due to the fact that their renin levels are several orders of magnitude above those in rats and humans. Thus, lowering circulating AGT in rats, combined with substantial renin upregulation, creates a mouse-like circulating RAS with still the same Ang II levels. This demonstrates the enormous versatility of the circulating RAS. Yet, combining AGT siRNA with an ARB (valsartan) caused an even further upregulation of renin, and under such conditions, accelerated consumption of AGT by renin resulted in the virtual disappearance of Ang II and a synergistic drop in blood pressure, that is, more than double the combined effects of siRNA and valsartan when given alone (Figure 3).^17^

The hypotensive effects of siRNA alone were identical to those of either the ACE inhibitor captopril or valsartan alone,^17^ and given that they were reached in the absence of changes in circulating Ang II, they most likely represented effects at the ‘local’ level. Indeed, AGT siRNA alone did lower renal Ang II (Figure 3) and virtually abolished renal Ang II in combination with valsartan.^17^ These data illustrate the sensitivity of the kidney (and possibly other tissues, like the vascular wall and heart) to hepatic AGT suppression, and argue against a role for renal AGT synthesis. Indeed, although GalNAc-conjugated AGT siRNA did not affect renal AGT mRNA, it did result in almost complete depletion of renal AGT.^17^ This agrees with recent studies showing that hepatic knockout of AGT, but not renal AGT knockout, prevented renal Ang II generation.^14,37^ The greater renal sensitivity to AGT suppression may be related to the fact that the renal AGT supply relies entirely on a complicated AGT uptake process involving megalin, depending on (normally) very high circulating AGT levels in the K_m_ range.^38–40^ Dropping these levels by >95% hampers sufficient uptake and will affect the possibility of generating sufficient angiotensins at renal tissue sites. A similar situation has been observed in the heart of subjects with end-stage heart failure receiving left ventricular assist device.^35^ Their renin levels were so high (immediately cleaving the small quantities of AGT that were still left), that cardiac AGT became depleted, thus no longer allowing Ang II generation at cardiac tissue sites.

**Figure 4.**
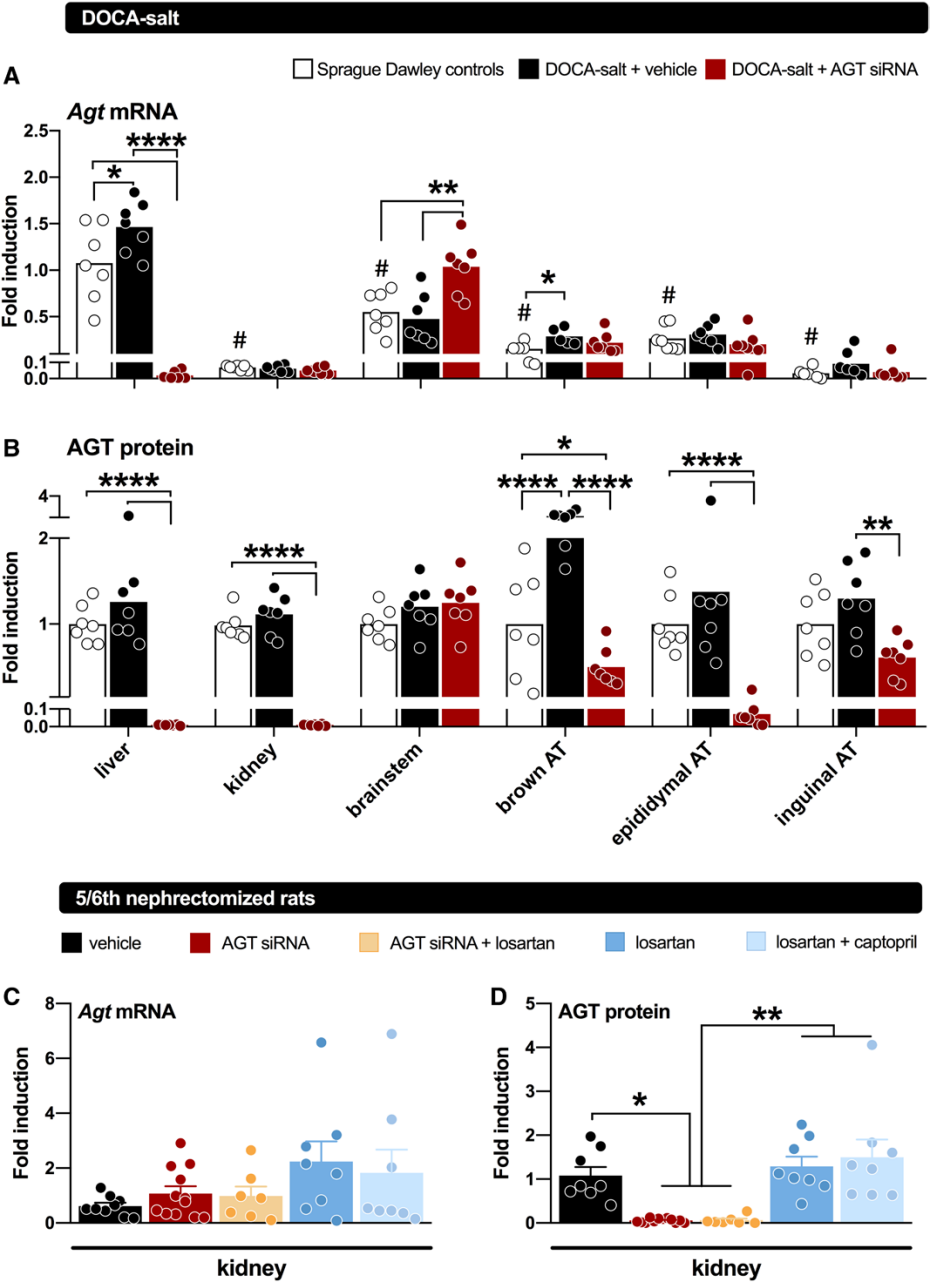
**Angiotensinogen (*AGT*) gene expression and protein abundance in tissues of rats. Top**, tissue *AGT* gene expression (represented as mean fold induction relative to *Agt* expression in the livers of non–deoxycorticosterone acetate [DOCA] controls) in rats not given DOCA-salt (non-DOCA) or given DOCA-salt for 7 wk and treated with vehicle or AGT small interfering RNA (siRNA) during the final 3 wk (n=7 per group). **Middle**, AGT protein abundance of AGT in tissues of non-DOCA and DOCA-salt–treated rats given vehicle or siRNA, represented as mean fold induction relative to AGT abundance in the non-DOCA controls of each tissue. AGT mRNA (normalized vs β_2_-microglobulin) and protein (fold induction vs GAPDH) levels in kidneys of Sprague-Dawley rats subjected to 5/6th nephrectomy, and treated with either vehicle, AGT siRNA, AGT siRNA+losartan, losartan, or losartan+captopril for 28 days (n=7–12). Treatment was started after 5 wk of recovery. Data are mean±SEM. AT indicates adipose tissue. *P<0.05, **P<0.01, ***P<0.001, ****P<0.0001. Data have been modified from Uijl et al^18^ and Bovée et al.^16^

AGT suppression has also been evaluated in low-renin hypertensive models, like the 8% salt-fed SHR,^28^ the 5/6th nephrectomy Sprague-Dawley rat,^16^ and the deoxycorticosterone acetate (DOCA) salt–treated Sprague-Dawley rat (Figure 3).^18^ In the former 2 models, its effects were identical to those of dual RAS blockade with an ACE inhibitor+ARB, whereas in the DOCA-salt model, it did not affect blood pressure. In the DOCA-salt model, hypertension is thought to rely on brain RAS activation.^41^ To what degree this truly occurs is controversial, given the absence of renin in the brain.^33^ Indeed, the small amounts of renin that could be detected in the brain (representing blood-derived, trapped renin) disappeared after DOCA-salt treatment, in parallel with the reductions in circulating renin after this treatment. As a consequence, brain Ang II levels, if anything, decreased after DOCA-salt.^18^ Nevertheless, liver-independent AGT synthesis did occur in the brain of the DOCA-salt Sprague-Dawley rat and was unaffected by liver-targeted AGT siRNA (Figure 4). Yet, AGT siRNA abolished brain Ang II in the DOCA-salt model.^18^ Since no effect on BP was observed, these data illustrate that brain Ang II in this model is unrelated to BP and that the synthesis of brain Ang II apparently relies on hepatic AGT. This raises the question of what the function of AGT synthesized at brain tissue sites is. Since brain-derived AGT is usually observed intracellularly,^42^ while angiotensin generation occurs extracellular (eg, in the interstitial space, or on the cell surface),^43^ one possibility is that it never reaches sites where it may react with renin (or even nonrenin enzymes), that is, that it has no function. A similar conclusion was reached with regard to adipose tissue AGT.^18,44^ Despite decades of research on its potential role,^5,8^ studies in both adipose tissue–specific AGT knockout mice and in rats treated with liver-targeted AGT siRNA (Figure 4) revealed that adipose tissue AGT after all is liver-derived.^18,29^ Hence, adipose tissue Ang II levels dropped after exposure to liver-targeted AGT siRNA.^18^ Indeed, Yiannikouris et al^29^ recently suggested that adipose tissue, like the kidney, sequesters circulating AGT via megalin.

In summary, AGT suppression lowers BP to the same degree as classical RAS blockers like ACE inhibitors and ARBs in a variety of hypertension models, and this effect is largely due to interference with tissue angiotensin generation. Unexpectedly, angiotensin generation at tissue sites where local AGT synthesis has been claimed to occur (mainly based on the demonstration of AGT mRNA), like the kidney, brain, and adipose tissue, appeared to rely fully on hepatic AGT, thus explaining the high efficacy of a liver-targeted approach. This raises the question of how hepatic AGT reaches these sites: possibly via simple diffusion into the interstitium, or by binding to a receptor, most likely megalin.

## AGT Suppression in Kidney Disease

Activation of the intrarenal RAS is an important contributor to the pathogenesis of hypertension and renal injury. Initially, an independent renal RAS was claimed to exist, relying on AGT synthesis at renal tissue sites, in particular at the level of the proximal straight tubule (S3 segment).^45^ Since Ang II stimulated AGT expression at this site, a so-called feed-forward loop was believed to contribute to kidney damage.^9–11^ However, we now know that tubular AGT expression, if occurring, at most results in the appearance of this AGT in urine, without contributing to renal Ang II production.^14,37^ Recent studies making use of hepatocyte-directed AGT siRNA in rats displaying hypertension and renal injury (SHR, DOCA-salt–treated hypertensive rats, and 5/6th nephrectomy rats; Figure 4) or liver-targeted AGT antisense oligonucleotides in normotensive cynomolgus monkeys all support this conclusion.^15–18^ AGT, like albumin, is reabsorbed from tubular fluid in the proximal tubules (S1 and S2 segments) via megalin, a multiligand receptor.^38^ Disruption of the glomerular filtration barrier increases delivery of AGT to the tubules and thus enhances kidney Ang II generation. In fact, when the glomerular barrier was disrupted by inducing podocyte injury, the amount of reabsorbed AGT is markedly increased, concurrently with an increase in urinary AGT.^14^ In this situation, megalin knockout further increased urinary AGT,^38^ and reduced the renal Ang II content. These results indicate that, in the nephrotic state, AGT filtered through the glomerulus and reabsorbed via megalin by the proximal tubule is converted to Ang II, while AGT in the tubular lumen is not. Moreover, megalin ASO greatly reduced renal Ang II in C57BL/6J mice, while simultaneously upregulating the urinary levels of renin and AGT.^39^ This demonstrates that also in normal mice, megalin is a determinant of renal angiotensin generation and that without megalin, filtered renin, and AGT are lost via urine. Similar observations have been made in patients with Dent disease or Lowe syndrome, in whom megalin-mediated reabsorption is disturbed.^40^

Urinary AGT has often been suggested to represent the renal RAS, assuming it is entirely derived from renal tissue sites.^46,47^ It now appears that major determinants of urinary AGT are rather glomerular filtration and megalin-mediated reabsorption, so that particularly under conditions where the glomerular barrier is disturbed, urinary AGT largely represents blood-derived hepatic AGT.^48^ Multiple factors determine megalin expression and function, including Ang II and urinary pH.^49^ Normally, AGT reabsorption is in the order of 95%, and thus a reduction to 90% or 85% might already double or triple urinary AGT levels. Hence, even small variations in megalin may cause great variation in urinary AGT levels for a given plasma AGT level, thus explaining why plasma and urinary AGT are not necessarily related. Nevertheless, given that urinary AGT is determined by 2 major renal parameters (filtration and reabsorption), while megalin-mediated reabsorption underlies renal Ang II generation, is not entirely unlikely that urinary AGT to a certain degree reflects kidney function and renal RAS activity.

Suppressing AGT in a chronic kidney disease model like the 5/6th nephrectomy rat prevented the development of proteinuria and reduced the occurrence of glomerulosclerosis.^16^ The reduction of renal Ang II (Figure 3) was an independent determinant of the former, together with blood pressure. AGT ASO decreased kidney size, cyst volume density, and blood urea levels in mouse models for polycystic kidney disease,^50,51^ and this was accompanied by a reduction in fibrosis and inflammation. These renoprotective effects in polycystic kidney disease were BP-independent, and although no angiotensin measurements were reported, it seems reasonable to assume that renal Ang II suppression was a major determinant of this outcome.

An important caveat of renal RAS suppression is that too much downregulation may deteriorate renal function and increase serum potassium. This is not surprising, given that Ang II is an important determinant of both glomerular filtration and adrenal aldosterone synthesis. Indeed, well-known consequences of too much RAS blockade are renal dysfunction (reflected by a rise in creatinine) and hyperkaliemia.^52^ Combined AGT siRNA-ARB treatment of SHR did upregulate serum potassium, yet without affecting glomerular filtration rate.^17^ Also in other hypertension models (5/6th nephrectomy and DOCA-salt), AGT siRNA, either alone or in combination with an ARB, did not reduce glomerular filtration rate.^16,18^ Clearly, more work is needed to carefully assess this aspect, particularly in diabetic kidney disease.

## AGT Suppression and Atherosclerosis

Atherosclerosis is a chronic, progressive disease manifested by lipid-laden macrophage and other leukocyte accumulation in lesions.^53^ The contribution of the RAS to atherosclerosis has been consistently demonstrated—ACE inhibitors and ARBs reduce lesion size and improve its cardiovascular outcomes in both human studies^54,55^ and animal models.^56^ Although the whole-body AGT deficient mouse was developed in the early 1990s,^30,57^ its severe growth and kidney development impairments impeded the use of this mouse for atherosclerosis studies.

An earlier study reported that leukocytes synthesize AGT.^58^ Although macrophages are the major cell type accumulated in mouse atherosclerotic lesions, bone-marrow transplantation of AGT deficient leukocytes did not reduce hypercholesterolemia-induced atherosclerosis in LDL (low-density lipoprotein) receptor^−/−^ mice,^59^ suggesting that leukocyte-derived AGT does not affect atherosclerosis. In contrast to the lack of effect of AGT in leukocytes, whole-body reduction of AGT in an AGT hypomorphic mouse model led to profound decreases in hypercholesterolemia-induced atherosclerosis,^59^ supporting that inhibition of AGT protects against atherosclerosis. Hepatocyte-specific deletion of AGT diminished atherosclerosis in LDL receptor^−/−^ mice fed a Western diet.^59–61^ Identical results were obtained with a liver-targeted AGT ASO (authors’ unpublished data). Collectively, these data from both genetic and pharmacological studies support the beneficial effects of selectively inhibiting liver AGT against atherosclerosis.

Hepatocyte-specific deletion of AGT reduces renal AGT protein and Ang II production,^39^ implicating that liver-derived AGT may contribute to atherosclerosis through augmenting renal Ang II production. Previous research suggested that the presence of Cys18-Cys138 disulfide bond in human AGT, representing oxidized AGT, regulates Ang II production.^62^ However, loss of oxidized AGT did not affect atherosclerosis in mice.^61^ Of note, plasma AGT in mice is exclusively oxidized,^61^ whereas the oxidized form accounts for ≈50% to 60% plasma AGT in humans.^62^ In addition, AGT is considered to be the rate-limiting component of the RAS in mice,^63^ but renin is the rate-limiting enzyme to regulate Ang II production in humans. Considering the many differences of AGT between humans and mice, it is necessary to determine whether the impressive atherosclerosis reductive effects by AGT inhibition in mice can be reproduced in humans or a human-like large animal model such as nonhuman primates.^15^ Most importantly, mouse atherosclerosis does not fully recapitulate the manifestations of human atherosclerosis.^64^ With the availability of human GalNAc AGT ASO and AGT siRNA,^26,65^ clinical trials on targeting liver AGT to treat human atherosclerotic disease are now feasible.

## AGT Suppression and Metabolic Disorders

In previous studies to determine effects of AGT on atherosclerosis, inhibition of AGT diminished Western diet–induced body weight gain and liver steatosis in mice.^59,66^ Mice with global genetic deletion of AGT,^67^ renin,^68^ or ACE^69^ have lower body weight and less fat attributed to impaired normal growth and kidney development, irrespective of being fed a normal laboratory diet or a fat-enriched diet. In contrast, inhibition of either whole-body or hepatocyte-derived AGT (genetic deletion or ASO) prevents Western diet–induced body weight gain and liver steatosis with normal growth and kidney function. Pharmacological inhibition of renin, ACE, or the AT_1_ receptor does not have these beneficial effects in mouse metabolism.^59^ Also, there is no solid evidence that ACE inhibitors and ARBs improve obesity or liver steatosis in humans.^70–72^ AGT is cleaved by renin into 2 products: one is Ang I (10 amino acids) that is further cleaved into Ang II (8 amino acids) by ACE, and the other product is des(Ang I)-AGT (443 amino acids in mice and 442 amino acids in humans). Adeno-associated viral vectors encoding either mouse AGT or des(Ang I)-AGT (ie, without Ang I-generating amino acids) with a liver-specific promoter were developed.^59^ Repopulation of either AGT or des(Ang I)-AGT in hepatocyte-specific AGT^−/−^ mice restored Western diet–induced obesity and liver steatosis, but only repopulation of AGT restored blood pressure and atherosclerosis comparable to wild-type mice.^59^ These data support that des(Ang I)-AGT contributes to obesity and liver steatosis in mice. Hepatocyte-specific AGT deletion increased energy expenditure and increased abundance of uncoupling protein 1 mRNA in brown adipose tissue, but did not affect food intake and physical activity.^59^ A recent study revealed that hepatocyte-specific deletion of AGT attenuates Western diet–induced alterations of the Akt (protein kinase B)/mTOR (complex 1 of the target of rapamycin)/SREBP-1c (sterol responsive element-binding protein 1c) pathway in liver, associated with liver steatosis.^66^ However, the definitive molecular mechanisms by which AGT contributes to obesity and liver steatosis have not been fully defined. In particular, it is unclear whether these metabolic benefits of inhibiting AGT will occur in humans.

## AGT Suppression and the Heart

Cardiac hypertrophy often accompanies high blood pressure. Lowering blood pressure will therefore prevent cardiac hypertrophy. However, since Ang II is an important profibrotic and growth factor, it is also possible that a reduction in cardiac hypertrophy occurs in a blood pressure-independent manner, via suppression of cardiac Ang II formation. Such formation depends on the uptake of circulating AGT.^31,35,73^ Indeed, in DOCA-salt hypertension, AGT siRNA did not affect blood pressure, yet did reduce the heart weight/tibia length ratio, a marker of cardiac hypertrophy.^18^ Since this effect was accompanied by the disappearance of cardiac Ang II, this likely represents the fact that AGT siRNA is capable of blocking Ang II formation at cardiac tissue sites. Although similar reductions in cardiac Ang II occurred in both SHR and 5/6th nephrectomy rats, the decrease in cardiac hypertrophy in these models correlated strongly with the decrease in blood pressure, suggesting that it was largely the consequence of blood pressure reduction.^16,17^

Sepsis is an acute and deadly health condition that triggers complex inflammatory responses. Ang II is used for vasodilatory septic shock,^74^ whereas ARBs may benefit septic patients with high arterial pressure.^75^ Recently, Rong et al^76^ reported that inhibiting liver-derived AGT improves sepsis-induced myocardial dysfunction. The authors found that deletion of AGT in the liver led to reduction of AGT protein in the heart, whereas cardiac-specific deletion of AGT did not change AGT protein abundance in heart. This confirms that liver-derived AGT supplies AGT protein in the heart. AGT protein is internalized via LRP1 (low-density lipoprotein receptor-related protein 1), a large protein in the LDL receptor superfamily, in cardiac fibroblasts. In vitro data support that AGT-LRP1 interaction promotes sepsis-induced myocardial dysfunction. Hepatocyte-specific deletion of AGT mitigates sepsis-induced myocardial dysfunction through both Ang II-dependent and independent mechanisms in mice. More studies are needed to establish the benefits of inhibiting AGT in sepsis and other infectious disease models.

## Human Data and Safety

Published human data on AGT suppression are scarce.^26^ Several Phase I/II trials are ongoing, which are expected to be finished over the next 1 to 2 years. IONIS-AGT-L_Rx_ (80 mg weekly via subcutaneous injection) has been evaluated in 2 double-blind, placebo-controlled trials in subjects with hypertension, either as monotherapy (25 patients) or as an add-on on top of ACE inhibitors/ARBs (26 patients) versus placebo for up to 2 months. The ASO reduced AGT by maximally 60% to 70%, and no changes in potassium levels, or liver and renal function were noted. Although not powered for this end point, trends were noted in blood pressure reduction. These data show that IONIS-AGT-L_Rx_ can lower AGT with a favorable safety profile. Based on these data, 2 12-week phase II, placebo-controlled trials are now scheduled to study IONIS-AGT-L_Rx_ in hypertension (ASTRAAS) and heart failure (ASTRAAS-HF). ASTRAAS will evaluate IONIS-AGT-L_Rx_ in 136 participants with uncontrolled blood pressure on top of 3 or more antihypertensive drugs (https://www.clinicaltrials.gov; Unique identifier: NCT04714320). ASTRAAS-HF will test the effect of IONIS-AGT-L_Rx_ on AGT and N-terminal prohormone of B-type natriuretic peptide in 72 chronic heart failure participants with reduced ejection fraction (https://www.clinicaltrials.gov; Unique identifier: NCT04836182). Zilebesiran is being tested in the placebo-controlled trials KARDIA-1 and KARDIA-2. KARDIA-1 (https://www.clinicaltrials.gov; Unique identifier: NCT04936035) will evaluate the effect of multiple doses of zilebesiran on systolic and diastolic blood pressure and characterize its pharmacodynamic effects and safety in 375 patients with mild-to-moderate hypertension. KARDIA-2 (https://www.clinicaltrials.gov; Unique identifier: NCT05103332) evaluates the efficacy and safety of zilebesiran as add-on therapy in 800 patients with hypertension that are not adequately controlled by standard of care antihypertensive medication (olmesartan, indapamide, or amlodipine).

Given the liver accumulation of the siRNA, one might speculate about liver toxicity, or inflammatory and immunologic side effects. Yet, no such observations were made for liver-targeted PCSK9 siRNA (inclisiran) over a 6-month period.^77^ Since this siRNA relies on the same GalNac principle, at present the safety profile of such drugs seems excellent. An important consideration will be how complete AGT suppression in humans needs to be, given the capacity of the body to upregulate renin. Normally, AGT levels are relatively stable, rising only during pregnancy (up to 2–3-fold),^78^ and decreasing significantly during huge renin upregulation, like in subjects with heart failure.^35,79^ Women display higher AGT levels than men, but this is counterbalanced by their lower renin levels.^80,81^ Similarly, the AGT M235T polymorphism-related changes in plasma AGT (carriers of the T allele displaying 5% to 10% higher AGT levels) are neutralized by inverse changes in renin (ie, carriers of the T allele displaying 5% to 10% lower renin levels),^82^ leaving plasma renin activity unaltered. To explain why the T allele still associates with multiple cardiovascular disorders,^83^ one has to assume, as discussed earlier, that such AGT variations somehow affect tissue angiotensin generation. This will also apply to AGT suppression by ASO or siRNA, and thus even modest reductions in AGT may have long-term beneficial consequences.

Clearly, although on the one hand the long-lasting effects of the ASO/siRNA approach might be considered as an advantage, circumventing compliance problems, it also poses a threat, for instance in women becoming pregnant during treatment, or in cases of emergency, when severe hypotension occurs, and the RAS is needed. Here novel tools (REVERSIR) are now being developed, capable of acutely reversing the effect of siRNA.^84^ This concerns short, synthetic, high-affinity oligonucleotides complementary to the siRNA guide strand that can be targeted to the liver making use of the same GalNac approach. An alternative might be fludrocortisone.^85^ It seems unlikely that complete AGT suppression should be the goal (although this is certainly feasible), given the well-known serious consequences of too much RAS blockade, observed for instance during dual or triple RAS blockade. A further possibility is the combination of a modest suppression of AGT with a second classical RAS blocker, which is likely to result in synergistic blood pressure lowering effects.^17^ The ongoing phase I/II trials will address these questions.

## Conclusions

AGT suppression in hepatocytes by ASO or siRNA is an exciting new tool to target the RAS with higher specificity and less adverse effects. It offers multiple new possibilities, either alone or in a synergistic combination with a second RAS blocker, to treat hypertension, kidney disease, or heart failure, acting particularly at the tissue level. Its long-term action (potentially requiring only 2 injections per year) might revolutionize pharmacotherapy and overcome compliance problems. Nevertheless, this also poses a threat in situations where the RAS is acutely needed, and thus preclinical and clinical programs are now carefully investigating its efficacy and safety profile, allowing an optimal introduction of AGT suppression as a novel treatment of cardiovascular and renal diseases in due time.

## Article Information

### Sources of Funding

The authors’ AGT (angiotensinogen)-related research work is supported by National Heart, Lung, and Blood Institute of the National Institutes of Health under award numbers R01HL139748 (H.S. Lu) and R00HL145117 (C. Wu). The content in this commentary is solely the responsibility of the authors and does not necessarily represent the official views of the National Institutes of Health. E.O. Cruz-López was supported by the Mexican National Council of Science and Technology (grant no. 739513).

### Disclosures

A.H.J. Danser received grant support from Alnylam Pharmaceuticals. The other authors report no conflicts.
